# RNA sequencing from human neutrophils reveals distinct transcriptional differences associated with chronic inflammatory states

**DOI:** 10.1186/s12920-015-0128-7

**Published:** 2015-08-27

**Authors:** Kaiyu Jiang, Xiaoyun Sun, Yanmin Chen, Yufeng Shen, James N. Jarvis

**Affiliations:** Department of Pediatrics, State University of New York at Buffalo School of Medicine, Buffalo, NY USA; JP Sulzberger Columbia Genome Center, Columbia University Medical Center, New York, NY USA; Departments of Systems Biology and Biomedical Informatics, Columbia University Medical Center, New York, NY USA

## Abstract

**Background:**

The transcriptional complexity of mammalian cells suggests that they have broad abilities to respond to specific environmental stimuli and physiologic contexts. These abilities were not apparent a priori from the structure of mammalian genomes, but have been identified through detailed transcriptome analyses. In this study, we examined the transcriptomes of cells of the innate immune system, human neutrophils, using RNA sequencing (RNAseq).

**Methods:**

We sequenced poly-A RNA from nine individual samples corresponding to specific phenotypes: three children with active, untreated juvenile idiopathic arthritis (JIA)(AD), three children with the same disease whose disease was inactive on medication (CRM), and three children with cystic fibrosis (CF).

**Results:**

We demonstrate that transcriptomes of neutrophils, typically considered non-specific in their responses and functions, display considerable specificity in their transcriptional repertoires dependent on the pathologic context, and included genes, gene isoforms, and long non-coding RNA transcripts. Furthermore, despite the small sample numbers, these findings demonstrate the potential of RNAseq approaches to biomarker development in rheumatic diseases.

**Conclusions:**

These data demonstrate the capacity of cells previously considered non-specific in function to adapt their transcriptomes to specific biologic contexts. These data also provide insight into previously unrecognized pathological pathways and show considerable promise for elucidating disease and disease-state specific regulatory networks.

**Electronic supplementary material:**

The online version of this article (doi:10.1186/s12920-015-0128-7) contains supplementary material, which is available to authorized users.

## Background

Neutrophils are the most common leukocytes in the human circulation and an important sentinel for recognizing invading micro-organisms and tissue damage. Thus, they are an important component of the acute response to infection and tissue injury. However, in recent years, we have also demonstrated that neutrophils show transcriptional aberrations in chronic childhood inflammatory diseases, including juvenile idiopathic arthritis (JIA) [1] and juvenile dermatomysositis [2]. In JIA, these transcriptional aberrations do not correct with therapy [3] and are associated with specific perturbations in cellular metabolic function [1]. Thus, in addition to their role in acute infectious and inflammatory disease, neutrophils appear to play important roles in chronic, indolent human inflammatory diseases.

The gene expression data used to elucidate the above findings were generated using conventional hybridization-based gene microarrays. The limits of hybridization-based microarrays are well documented [4]. Furthermore, hybridization-based arrays fail to capture the full complexity of the transcriptome, including novel alternatively spliced isoforms and non-coding RNAs. Therefore, gene microarrays have serious limits from the standpoint of understanding the transcriptional-rewiring [5] that very likely underlies many complex human diseases.

RNA sequencing techniques carry the promise of revolutionizing our understanding of the transcription processes that underlie phenotypes [6]. As data from projects like ENCODE [7] reveal the complexities of the transcriptome in eukaryotic cells, it is becoming clear that, in order to fully understand human pathological cellular networks, we are going to need more detail of the transcriptional events that underlie disease phenotypes.

Neutrophils are a particularly challenging cell with which to work. The presence of endonucleases within human neutrophils, a part of the host defense against bacteria [8], presents particular challenges to preparing high-quality nucleic acid for sequencing studies. Neutrophils are thus conspicuously absent from both the ENCODE and Roadmap Epigenomics data sets. The studies we report here were undertaken to determine the specificity of neutrophil transcriptomes to specific human illnesses or disease states, a prerequisite for biomarker development, by examining specific phenotypes that show subtle differences from one another.

## Methods

### Patients and patient samples

Neutrophils were collected from nine children after informed consent was obtained from their parents according to a protocol approved by the University of Oklahoma Health Sciences Center Institutional Review Board. Three of the samples were from children (ages 5–10 years, all girls) with newly-diagnosed, untreated polyarticular juvenile idiopathic articular arthritis (JIA). Samples were also obtained from 3 patients; also girls aged 5–10, who fit criteria for clinical remission on medication (CRM). That is, these children had normal physical exams, no symptoms of arthritis (morning stiffness, gait disturbance, fatigue) and normal laboratory studies (complete blood counts, erythrocyte sedimentation rate) and had maintained this state for at least 6 continuous months. In addition, a control population consisting of 3 children with cystic fibrosis (CF) (ages 6–21 years, all boys) was also studied. The latter group is an important and seldom used-control; children with CF have chronic, indolent inflammation in the lung, and thus allow us to discern disease-specific characteristics in JIA from those that might be seen in any chronic, sub-acute inflammatory state. Children with CF were seen during routine follow-up and were stable from the standpoint of pulmonary symptoms at the time they were studied.

### Cell isolation

Whole blood was drawn into 10 mL CPT tubes (Becton Dickinson, Franklin Lakes, NJ), which is an evacuated blood collection tube system containing sodium citrate anticoagulant and blood separation media composed of a thixotropic polyester gel and a FICOLL™ Hypaque™ solution. Cell separation procedures were started within 1 h from the time the specimens were drawn. Neutrophils were separated by density-gradient centrifugation at 1,700× g for 20 min. After removing red cells from neutrophils by hypotonic lysis, neutrophils were then immediately placed in TRIzol® reagent (Invitrogen, Carlsbad, CA) and stored at −80 °C until used for RNA isolation. Cells prepared in this fashion are more than 98 % CD66b + by flow cytometry and contain no contaminating CD14+ cells, as previously reported [9]. Thus, although these cell preparations contained small numbers of other granulocytes, they will be referred to here as “neutrophils” for brevity and convenience.

### RNA isolation and sequencing

Total RNA was extracted using Trizol® reagent according to manufacturer’s directions. RNA was further purified using RNeasy MiniElute Cleanup kit including a DNase digest according to the manufacturer’s instructions (QIAGEN, Valencia, CA). RNA was quantified spectrophotometrically (Nanodrop, Thermo Scientific, Wilmington, DE) and assessed for quality by capillary gel electrophoresis (Agilent 2100 Bioanalyzer; Agilent Technologies, Inc., Palo Alto, CA). Single-end cDNA libraries were prepared for each sample and sequenced using the Illumina TruSeq RNA Sample Preparation Kit by following the manufacture’s recommended procedures and sequenced using the Illumina HiSeq 2000. Library construction and RNA sequencing were performed in the Columbia Genome Center in Columbia University Medical Center.

### Data processing and analysis

The short reads were mapped to the reference genome (Human: NCBI/build37.2) using TopHat (version 2.0.4) [10] with 4 mismatches (−−read-mismatches = 4) and 10 maximum multiple hits (−−max-multihits = 10). Transcripts were assembled and the relative abundance (aka expression level) of genes and splice isoforms were estimated using Cufflinks in “fragments per kilobase of exon model per million mapped reads” (FPKM) [11]. (version 2.0.2) with default settings. Differential expression genes and exomes were tested using DEseq. To define significantly differential expression genes/exomes, we used a *p*-value < 0.05 as the cutoff. The Database for Annotation, Visualization and Integrated Discovery (DAVID), v6.7, (http://david.abcc.ncifcrf.gov/home.jsp) was used for Gene Ontology (GO) analysis.

### Ingenuity Pathway Analysis (IPA)

To identify upstream regulators of the differentially expressed genes between AD and CRM or between AD and CF, we used IPA software (Ingenuity Systems, Redwood City, CA). Gene symbols were used as identifiers and the Ingenuity Knowledge Base gene set as a reference for a pathway analysis. Identification of upstream transcription regulator was assessed using IPA where the activation or inhibition of a transcription regulator was determined from expression patterns of the transcription factor and its downstream-regulated genes within the differentially expressed list. The absolute value of the z-score ≥ 2.0 was considered statistically significant with a positive value indicating activation and a negative values indicating inhibition of the transcription factor.

### Differentially expressed genes and LncRNA expression validation by quantitative real-time RT-PCR

Total RNA was reverse transcribed with iScript™ cDNA synthesis kit according to the directions of the manufacturer (Bio-Rad, Hercules, CA, USA). Real-time RT-PCR was performed using SYBR Green reagents on a StepOne Plus (for the testing group; Applied Biosystems, Foster City, CA, USA) as described previously [3]. Gene-specific amplification was confirmed by a single peak in the ABI Dissociation Curve software. Average threshold cycle (Ct) values for GAPDH (run in parallel reactions to the genes of interest) were used to normalize average Ct values of the gene of interest. These values were used to calculate averages for each group, and the relative ΔCt was used to calculate fold-change values between the groups. The nucleotide sequences of the primers are listed in Table 1. All primers applied were tested to display an efficiency of amplification approximate 98 % (±SD 4.65 %).

**Table 1:** Primers used for real-time PCR and real-time PCR validation of RNA-seq results

Gene ID	Primer direction	Primers sequence (5′ ~ 3′)	Fold change (AD vs CRM)	
			RNA-seq	qPCR
DDX60	Forward	GAA GCA GCA GGA AGC TGA A	−5.73	−1.26
	Reverse	GGA TGT CTC TCA GTT GCT CAA A		
IFIH1	Forward	TTG GAT AAG TGC ATG GAG GAG	−3.61	−1.53
	Reverse	CCT GTT TGA CGA AGA ACA TTC AG		
IFITM3	Forward	CCT GTT CAA CAC CCT CTT CA	−5.30	−1.82
	Reverse	CAT GAG GAT GCC CAG AAT CA		
IGHMBP2	Forward	ACG AAC AGT CGA AAG GGA AC	2.99	1.10
	Reverse	AGC CAT CGA CAG ACT TGA TTT		
MOV10	Forward	GGG CTA TGA CCT GGA GTT AAG	1.44	−3.50
	Reverse	CAC CTC ATA GTT CCT CCA CTT C		
OAS1	Forward	GAA GCC TGT CAA AGA GAG AGA G	−13.46	−1.24
	Reverse	GTT AGG TTT ATA GCC GCC AGT		
PML	Forward	ACA ACA TCT TCT GCT CCA ACC	−2.64	2.21
	Reverse	TGT CGC TGC TGG ATC TCT		
RNF213	Forward	CTG GTT GTG TCA CCT CCT AAC	−3.07	1.46
	Reverse	GTC CTT GTG TCC ATG CAT CT		
TNFAIP6	Forward	GAT GGG ATG CCT ATT GCT ACA	−2.79	−3.70
	Reverse	CGC TGA CCA TAC TTG AGT CTA AT		
TRIM5	Forward	GCA GGA AGC TGA AGA GTT AGA	−1.33	−2.58
	Reverse	GAA TGT CTT CCT CCT CCT TCT C		
lnc-CKAP2L-1	Forward	GTTAAAGCTGCGAAGAACCTAAC	13.94	1.19
	Reverse	TTCCTGCCTCTTCCTACTCT		
lnc-IFITM2-4	Forward	GATCTTAGCCTTGGCCTCAC	−7.98	−24.85
	Reverse	TACACCAGGCAACCACAAATA		
lnc-IRS2-2	Forward	GCTAGTTCAGCCTGTGAGATG	17.2	6.69
	Reverse	AGCAAGCAATCCAAGAGAGAG		
lnc-PFDN4-1	Forward	GGTGTTTGGAGACAAAGGAATAG	3.77	1.28
	Reverse	CTATCTCGTGCCGCTTAGTATC		
lnc-PML-1	Forward	TGTAGCACTCACGGCAAAT	−6.16	−2.39
	Reverse	CGTGTCCAGAGTTTGTTCCT		
lnc-RBL2-1	Forward	TCCTGAGTAGCTGGGAT GTA	−2.2	−1.61
	Reverse	GACCAGCCTAGCCAACATAAT		
lnc-SLC2A13-1	Forward	TAATGGCAGTGGAGGTTGTC	−2.78	−3.39
	Reverse	GAACTTCCAGCATCTCCTTACA		

## Results

### An overview of RNA-seq data

In this study, we conducted genome-wide RNA sequencing for 9 individual samples corresponding to specific phenotypes: 3 children with active, untreated juvenile idiopathic arthritis (JIA), a common chronic disease in children characterized by inflammation and hypertrophy of synovial membranes [12, 13]. These subjects will be referred to as AD. We also studied 3 children with the same disease whose disease had been inactive and stable for 6 continuous months on the anti-inflammatory, immunosuppressive drug, methotrexate. The children were described as being in clinical remission on medication (CRM) as defined by accepted international criteria [14]. Finally, we studied 3 children with cystic fibrosis (CF), an autosomal recessively inherited disorder characterized by chronic, indolent inflammation in the lungs. For all 3 AD samples, we generated an average of 19.35 million 101 bp reads per sample, and average number of reads mapped to the genome was 13.59 millions. For the CRM samples, the average number of reads per sample was 20.82 million, and average number of reads mapped to the genome was 14.34 million. For the CF samples, an average number of reads per sample was 20.53 million, and the average number of reads mapped to genome was 16.84 millions (Table 2). There were no significant differences in the number of reads between AD and CRM or CF samples. The sequencing performance and library quality was further assessed using RNA-SeQC v1.1.7 [15]. The results show that 76.8 % of reads mapped to known exons, and ~18.7 % mapped to intronic regions. These statistics indicate that the sequencing data is robust (Table 3).

**Table 2:** RNA-Seq sequences reads mapping to NCBI human genome build37.2 by TopHat (version.2.0.4)

Sample name	Number of raw reads	Number of mapped reads	Mapped reads %
AD 1	21,363,317	14,752,865	69.06
AD 2	14,740,041	10,415,236	70.66
AD 3	21,940,589	15,601,068	71.11
Average	19,347,982	13,589,723	70.27
CRM 1	22,302,984	15,579,477	69.85
CRM 2	18,410,947	12,278,180	66.69
CRM 3	21,735,005	15,170,922	69.80
Average	20,816,312	14,342,859	68.78
CF 1	16,607,430	13,228,449	79.65
CF 2	20,084,504	16,607,129	82.69
CF 3	24,909,069	20,696,538	83.09
Average	20,533,668	16,844,038	81.81

**Table 3:** RNA-SeQC analysis of sequencing performance and library quality

Sample name	Intragenic rate	Exonic rate	Intronic rate	Intergenic rate	Expression profiling efficiency	Transcripts detected	Genes detected
AD 1	0.954	0.758	0.196	0.046	0.758	85,660	15,787
AD 2	0.949	0.727	0.223	0.05	0.727	76,885	14,478
AD 3	0.956	0.78	0.176	0.043	0.78	72,871	13,783
CRM 1	0.956	0.766	0.19	0.043	0.766	81,034	15,094
CRM 2	0.953	0.767	0.186	0.046	0.767	72,810	13,772
CRM 3	0.956	0.78	0.176	0.044	0.78	77,082	14,628
CF 1	0.957	0.767	0.19	0.043	0.767	75,374	14,245
CF 2	0.958	0.78	0.178	0.041	0.78	85,761	16,101
CF 3	0.956	0.785	0.171	0.043	0.785	71,900	13,552

### Gene expression analysis

Overall, there were 12,050 genes expressed in neutrophils in at least one of nine subject with FPKM value >1. Of these, 7734 genes identified as expressed in neutrophils were detected in all nine subjects (Additional file 1: Table S1). As expected, these genes include transcripts for cytokines/chemokines, cell-surface receptors, major histocompatibility complex (MHC) proteins, apoptosis regulators and adhesion molecules, and proteases, all important to neutrophil function. In order to further characterize these genes, we classified genes into three groups based on their FPKM values: high expression (top 25th percentile; FPKM > 36.84), medium expression (middle 50^th^ percentile; 5.63 < FPKM ≤ 36.84), and low expression (bottom 25^th^ percentile; FPKF ≤ 5.63). We then carried out Gene Ontology (GO) analysis using the Database for Annotation, Visualization and Integrated Discovery (DAVID), v6.7, http://david.abcc.ncifcrf.gov/home.jsp [16]. GO analysis is an additional useful bioinformatics tool to categorize and group large gene sets based on a known functional associations, as defined by the Gene Ontology Consortium [17]. This analysis revealed that high expression genes are enriched for translational elongation (*p* = 6.90E-46, FDR = 1.28E-42), immune responses (*p* = 8.73E-26, FDR = 1.62E-22), defense responses (*p* = 1.75E-20, FDR = 3.25E-17), intracellular signaling cascades (*p* = 3.03E-17, FDR = 5.63E-14), and inflammatory responses (*p* = 2.54E-14, FDR = 4.69E-11). Medium-expression genes were enriched for transcripts involved in protein catabolic processes (*p* = 2.00E-28, FDR = 3.74E-25), cellular macromolecule catabolic processes (*p* = 2.10E-28, FDR = 3.94E-25), and cellular protein catabolic process (*p* = 4.93E-28, FDR = 9.24E-25). Low-expression genes are involved in transcription (*p* = 6.52E-11, FDR = 1.17E-07) and DNA metabolic processes (*p* = 9.66E-09, FDR = 1.73E-05). The top 5 categories of the GO analysis for 3 groups are presented in Table 4. All categories are presented in Additional file 2: Table S2.

**Table 4:** Gene ontology analysis of genes expressed in neutrophils

Category	Term	Count	*P* value
High expression genes			
GO:0006414	Translational elongation	72	6.90E-46
GO:0006955	Immune response	169	8.73E-26
GO:0006952	Defense response	145	1.75E-20
GO:0006412	Translation	91	2.00E-17
GO:0007242	Intracellular signaling cascade	230	3.03E-17
Medium expression genes			
GO:0030163	Protein catabolic process	238	2.00E-28
GO:0044265	Cellular macromolecule catabolic process	266	2.10E-28
GO:0051603	Proteolysis involved in cellular protein catabolic process	231	4.93E-28
GO:0043632	Modification-dependent macromolecule catabolic process	223	1.07E-27
GO:0019941	Modification-dependent protein catabolic process	223	1.07E-27
Low expression genes			
GO:0006350	Transcription	268	6.52E-11
GO:0006974	Response to DNA damage stimulus	70	2.42E-09
GO:0006259	DNA metabolic process	85	9.66E-09
GO:0006281	DNA repair	56	2.03E-08
GO:0045449	Regulation of transcription	302	8.31E-08

Genes were classified into three groups, high-expression (25 %), medium-expression (50 %) and low expression (25 %). Gene ontology analysis was performed using DAVID. Top 20 categories for each group was presented here

**Fig. 1:**
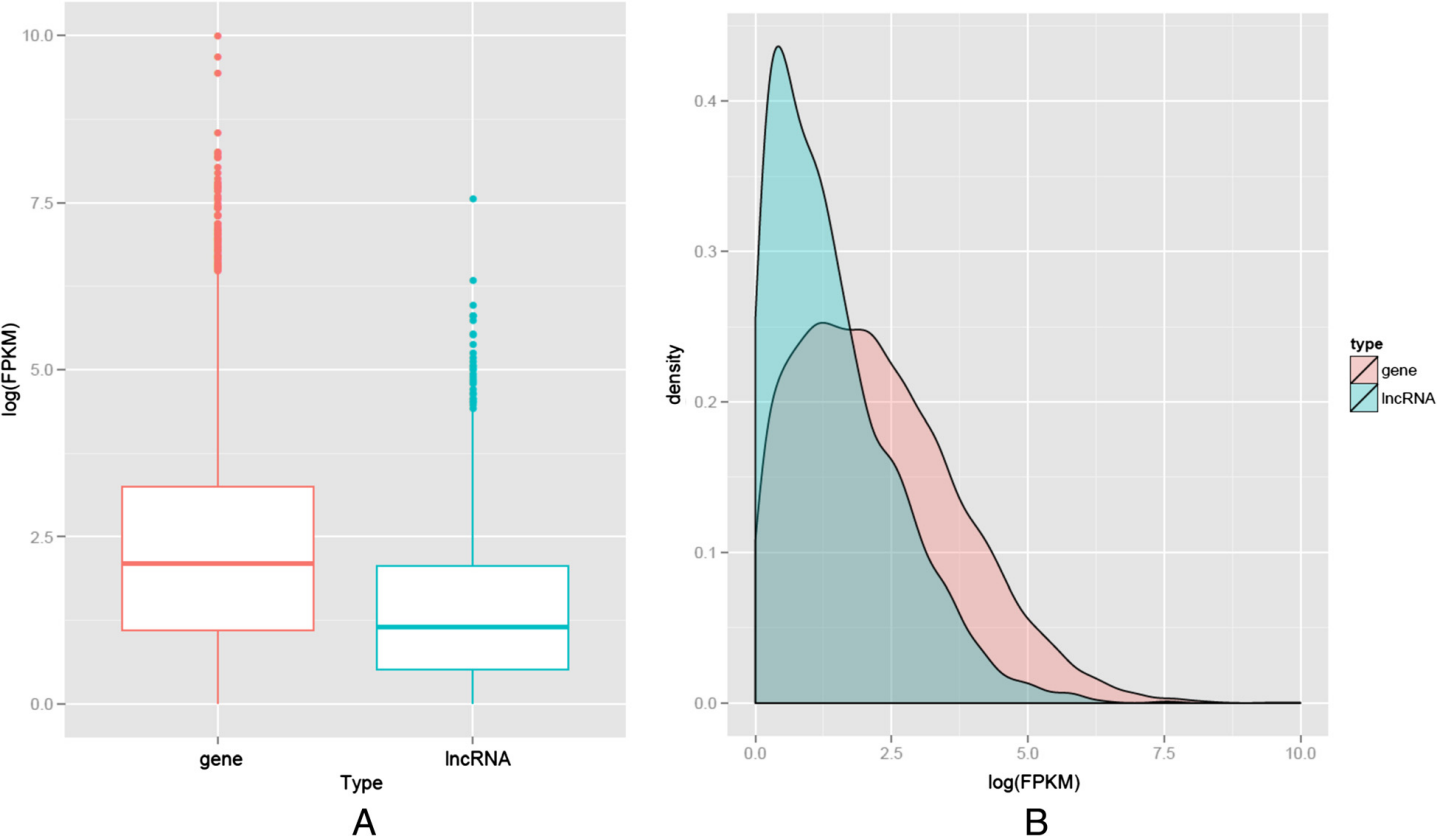
A comparison of expression level of protein coding genes and non-coding transcripts. The expression levels of non-coding transcript were lower than of protein coding genes. **a** the box plot indicates expression level (log FPKM) of all protein-coding genes in neutrophils as compared to that expression level of all non-coding genes in neutrophils. **b** Kernel density plot of FPKM in log scale for protein coding genes and lncRNAs

Long non-coding RNAs (lncRNAs) are defined as transcripts of greater than 200 nucleotides without evident protein coding function [18]. Thus, lncRNA is a broad definition that encompasses multiple different classes of RNA transcripts, including enhancer RNAs, small nucleolar RNA (snoRNA) hosts, intergenic transcripts, and transcripts overlapping other transcripts in either sense or antisense orientation. So far, only a few RNA-Seq studies have detected or analyzed lncRNAs [19]. We took advantage of our next generation RNA-Seq data to identify all lncRNA expressed in neutrophils. We mapped the RNA-seq data to a comprehensive compendium of long non-coding RNAs (www.LNCipedia.org, version 2.0). The current version of this long non-coding RNA database contains 32,183 human annotated lncRNAs [20]. We found 2981 lncRNAs were expressed in neutrophils in at least one of nine subjects (FPKM ≥ 1) (Additional file 3: Table S3). As expected, these non-coding transcript FPKM values were lower than protein coding genes (Fig. 1). Multiple studies have shown that lncRNA expression details vary for different classes [21, 22] and there is evidence that lncRNAs participate in multiple networks regulating gene expression and function [23]. LncRNAs also interact with multiple proteins, enabling scaffolding functions and combinatorial control [24]. For example, the recently identified lincRNA-Cox2 mediates both the activation and repression of distinct classes of immune genes [25]. However, the *in vivo* functions of most of the currently annotated lncRNAs have not been determined, including the 2981 lncRNA we detected in neutrophils.

We used Cufflinks to assemble transcripts and estimate the abundance of isoforms. There are 31,046 isoforms expressed in neutrophils in at least one of nine subjects with FPKM value >0 (Additional file 4: Table S4). While the genes have several alternatively spliced transcripts, their isoforms are not expressed at equivalent levels. For most genes, one isoform is expressed more highly than others (data not shown), a finding compatible with what has been reported in B cells [11].

### Identification of differentially expressed genes between phenotypes

Although neutrophils are typically regarded as non-specific mediators of inflammatory responses, data from hybridization-based gene expression arrays suggest that there may be subtle differences in neutrophil transcriptomes that correlate with human disease phenotypes [26]. We used DESeq [27] to test the differential expression of genes comparing the three phenotypes. When the expressed genes are defined as FPKM ≥1 in all 3 replicates, there are 8597 expressed genes in the AD neutrophils, 8668 expressed genes in CRM neutrophils and 8102 expressed genes in the CF neutrophils, which were aligned to the reference genome (data no shown).

One hundred fifty-nine genes showed differential expression when children with active juvenile arthritis (the AD group) were compared to children with sustained, inactive disease on medication (the CRM group - Additional file 5: Table S5). Twenty of these genes were expressed in higher levels in AD and 136 were expressed in lower levels in AD compared with CRM (top 10 up and down regulated DE genes in Table 5). There are two genes (*ADARB2, C20orf134*) only expressed in AD and one gene, *FLJ31813* was expressed only in CRM. The *ADARB2* gene (RNA-specific adenosine deaminases, B2) is associated with brain tumors [28] and a single nucleotide polymorphism (SNP) in *ADARB2* is associated with metabolic disorders [29]. An inflammatory/immune function for *ADARD2* is not known. Genes showing lower levels of expression in AD included *ICAM-1, IL-1B, CCR1, IFIH1, SOCS1, TNFAIP3 and TNFSF13B*, which are strongly associated with acute and chronic inflammation and adult rheumatoid arthritis. For example, SOCS1 was up-regulated in the synovial membranes from patients with RA when compared with osteoarthritis [30]. Interferon-induced proteins (*IFI35, IFI44, IFI44L, IFI6, IFIH1, IFIT2, IFIT3, IFIT5, IFITM1, IFITM3*) also show lower levels of expression in neutrophils of children with AD, suggesting that attenuated interferon responses may be an underlying aspect of the disease. In support of this idea was the finding that Type I IFN response genes *OAS1*, *OAS2* and *OAS3*, were also expressed in lower levels in AD samples compared with CRM. This finding corroborates studies that have shown that, at least in adult patients with rheumatoid arthritis, therapies with anti-TNF antibody, a common treatment for severe forms of the disease, induce significant increases in type I IFN response gene activity [31]. Other genes expressed in lower levels in AD are associated with immune responses or responses to viruses. The *C5orf56* gene also showed lower expression in AD. This gene is of special interest, as, in a recent GWAS for the juvenile arthritis phenotype studied here, SNPs within the *C5orf56* region had one of the strongest associations with genetic risk among the regions identified [32]. The *PAM* gene (Peptidylglycine alpha-amidating monooxygenase) was up-regulated in AD. *PAM* cleaves immature adrenomedullin (an antiapoptotic peptide) to the mature peptide. PAM also expressed rheumatoid arthritis fibroblast-like synoviocyte and osteoarthritis fibroblast-like synoviocyte [33].

**Table 5:** Top 10 up and down regulated differentially expressed genes in neutrophils in juvenile idiopathic arthritis with active status compared with in juvenile rheumatoid arthritis with clinical remission on medicine status

Gene symbol	Base Mean_all	Base Mean_AD	Base Mean_CRM	*p* value	Fold change (AD vs CRM)
LOC100652901	2.584163876	4.893556785	0.274770967	0.028780503	17.80958462
APOBEC3B	83.15003176	153.1234541	13.17660941	0.046805157	11.620854
AASS	4.098483224	7.372653548	0.824312901	0.03030685	8.943998745
ELF5	5.151781844	8.903353288	1.400210399	0.009241724	6.358582465
COL4A3	4.238990844	7.254099279	1.223882409	0.035842855	5.927121121
ZNF772	8.041388866	13.0963408	2.986436929	0.027467532	4.385272857
PAM	185.1146538	300.6639308	69.56537684	0.035123308	4.3220341
RNU5A-1	13.32454668	21.2148916	5.434201748	0.009970308	3.903957304
CEACAM19	12.44161404	19.01848534	5.864742738	0.035882882	3.242850743
LOC100128028	9.96808427	15.18599541	4.75017313	0.038699912	3.196935142
IFI44	1090.259979	234.9096707	1945.610288	0.034410811	−8.282376295
OAS3	3312.067396	599.3511189	6024.783673	0.012004606	−10.05217723
OAS2	1177.854158	208.429431	2147.278884	0.045495414	−10.30218657
PGM5	60.33985892	10.55582037	110.1238975	0.027057445	−10.43252856
IFI44L	1221.349953	203.5852067	2239.1147	0.013271076	−10.99841554
TIAF1	2.970579096	0.413585692	5.5275725	0.029142879	−13.36499935
OAS1	675.8399068	93.42104659	1258.258767	0.00911317	−13.46868626
LY6E	887.4348129	108.049892	1666.819734	0.022952433	−15.42638964
LILRB5	3.535606771	0.413585692	6.657627849	0.044736858	−16.09733601
FAM21B	36.36690516	4.133439531	68.60037079	0.022509698	−16.59643749

**Fig. 2:**
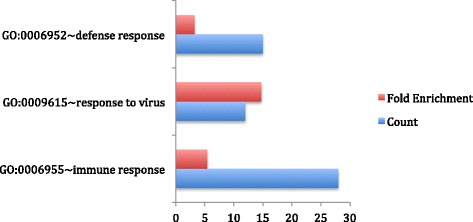
Functional enrichment analysis results for differentially expressed genes between AD and CRM

In order to characterize the differentially expressed genes, we conducted a Gene-Ontology analysis using DAVID as described above. Unsurprisingly, the top three functional groups enriched in differentially expressed genes are: (a) immune response (*p*-value = 4.34E-13, FDR = 7.06E-10), including 28 differentially expressed genes; (b) response to viral infection (*p*-value = 4.33E-10, FDR = 7.04E-7), including 12 genes, and (c) host defense responses (*p* value = 1.65E-4, FDR = 0.2682), including 15 genes (Fig. 2).

We further examined the specificity of neutrophil transcriptomes by examining another phenotype characterized by chronic, soft tissue inflammation. CF is an autosomal-recessively inherited disease caused by mutations in the *CFTR* gene that lead to abnormal ion transport in respiratory epithelial cells [34, 35]. These abnormalities are associated with chronic, indolent inflammation in the small airways and lung parenchyma, due, in part, to chronic infection/colonization with pseudomonas aerigninosa as well as other bacteria [36]. Lungs of affected patients show a characteristic neutrophilic infiltrate and high concentrations of tumor necrosis factor-alpha (TNF-a), which amplifies the inflammatory process by stimulating release of *IL-1, IL-6, IL-8* [37, 38]. CF thus presents a good control and comparison group to children with JIA, as it allows us to identify transcriptional reorganization that is disease-specific and distinguish it from changes that are generic to chronic inflammation in soft tissues.

Because the CF patients consisted entirely of males, while the JIA patients were females, we excluded genes on either sex chromosome that showed differential expression in the comparison of CF and JIA patients. There were 113 genes that showed differential expression with at least 1.9-fold change (*p* < 0.05) when neutrophils from children with untreated arthritis were compared with those from children with CF (Additional file 5: Table S5). Forty of these gene transcripts were found in greater abundance in the neutrophils from children with untreated arthritis and 70 were found in higher in the neutrophils from CF (top 10 up and down regulated DE genes in Table 6). There are two genes (*MYH6, PPP4R4*) expressed only in CF and gene *ELOVL2* expressed only in AD. Predictably, many of the DE genes are involved inflammatory responses (*KLRG1, CCR5, C4A, CCR4, CFD, SPP1*) and immune responses (*CCR5, C4A, IFITM2, CCR4, MSH2, FCGR2C, TREM1, CFD*). Interestingly, there are ten DE genes that are associated with cell cycle regulation (*CDKN1A, PLK3, ZC3HC1, MSH2, CYP26B1, KIF20B, CENPV, G0S2, CDK6, AHR*).

**Table 6:** Top 10 up and down regulated differentially expressed genes in neutrophils in juvenile idiopathic arthritis with active status compared with in neutrophils in cystic fibrosis

Gene symbol	Base Mean_all	Base Mean_AD	Base Mean_CF	*p* value	Fold change
					(AD vs CF)
HRK	3.254095483	6.295412043	0.212778924	0.00913228	29.58663351
ZNF724P	2.434400622	4.65602232	0.212778924	0.038928663	21.88197136
DUOX2	4.06892349	7.551846549	0.58600043	0.007728683	12.8871007
LOC145474	3.478432752	6.32498152	0.631883985	0.015249855	10.00971962
CFD	1673.012068	2996.168793	349.8553432	0.00893998	8.56402182
COL5A3	8.493207647	14.96975153	2.016663769	0.029568293	7.423027953
KIF21A	7.226812819	12.17829939	2.275326247	0.043226873	5.35233108
KLRG1	14.29693321	23.81752942	4.77633699	0.022752462	4.986568048
EFHA2	6.265641701	10.20362082	2.327662587	0.04898428	4.383633984
TRIM36	9.077531529	14.75361951	3.401443553	0.026681604	4.337458282
LOC285847	2.582363565	0.443745708	4.720981423	0.047502758	−10.63893428
PGM5	70.05844466	11.57358217	128.5433071	0.015904627	−11.10661377
LOC100133207	3.462096626	0.458530446	6.465662806	0.033308951	−14.10083641
LRRC32	2.183632721	0.229265223	4.138000219	0.042150439	−18.04896602
RPH3A	234.0009908	24.42986525	443.5721163	0.0438117	−18.15696123
FAM21B	44.9347236	4.54591393	85.32353326	3.54E-07	−18.76928041
PCOLCE2	3.287165275	0.229265223	6.345065326	0.021037444	−27.67565548
GLIS3	6.916159962	0.229265223	13.6030547	0.000110398	−59.33326704
CYP26B1	85.98114317	2.337885371	169.624401	4.17E-14	−72.55462697
TMTC1	285.5146376	6.717864103	564.3114112	0.002300462	−84.00161161

To confirm the differences in gene expression between AD and CRM observed in the RNA-seq experiments, we performed real-time qRT-PCR. Ten genes that showed significant differentially expressed between AD and CRM in the RNA-seq analysis (*DDX60, IFH1, IFITM3, IGHMBP, MOV10, OAS1, PML, RNF213, TNFAIP6, TRIM5*) were analyzed by real-time qRT-PCR in an independent patient cohort. Table 1 shows that seven of ten genes differentially expressed in the RNAseq analysis were also differentially expressed in the real-time qRT-PCR.

### Sample reproducibility

**Fig. 3:**
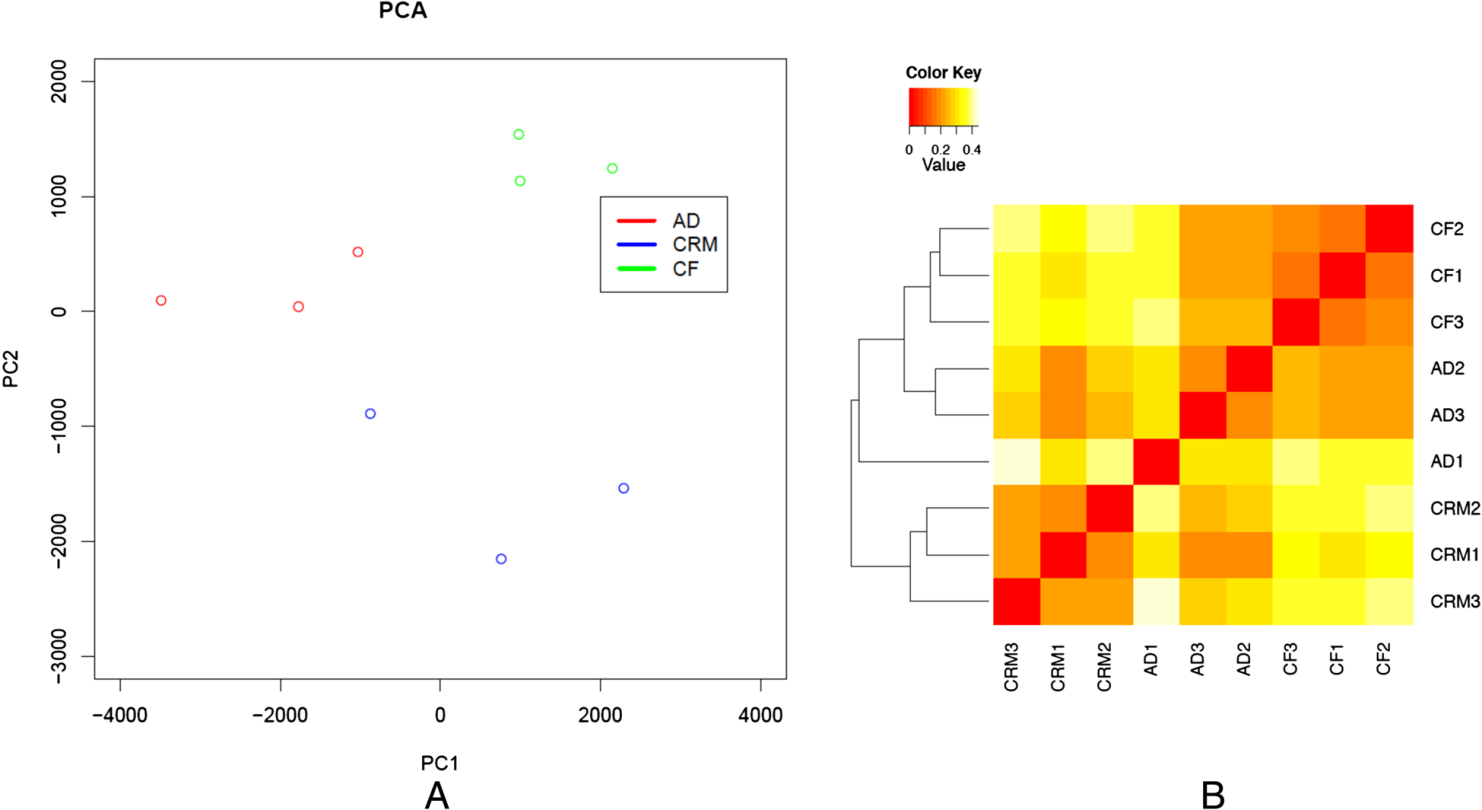
Sample reproducibility. **a** Principal Component Analysis (PCA) was performed based on differentially expressed genes (*p* < 0.05) to obtain an overview for the three conditions (AD, CRM, CF) on gene expression. The PCA scatter plot showed that the three groups (AD, CRM, CF) is the best separation and formed three distinct clusters. **b** Heat map of sample distance based on Jensen Shannon entropy over gene counts. The result showed three groups were able to be separated

To assess reproducibility of gene expression of biological represents in neutrophils, Principal Component Analysis **(**PCA) and sample pairwise correlation coefficient calculation are performed to obtain an overview of gene expression for the three conditions (AD, CRM, CF). A PCA scatter plot (Fig. 3a) and heat map of heat map of sample distance based on Jensen Shannon entropy over gene counts (Fig. 3b) show that the three groups (AD, CRM, CF) formed three distinct clusters. While the sample designated AD1 appears to be somewhat distinct in the heat map, this sample still clusters closest with the other 2 AD samples. This sample was from a 10 year old girl, while the other 2 were from 5 and 6 year old girls. It is possible that early pre-pubertal changes may have had an effect on gene expression in this patient. Thus, although the differences in the three phenotypes are relatively subtle from an immunologic standpoint, neutrophils in each show subtle specificity that suggests fine-tuning of the transcriptome in response to specific inflammatory environments, or, in the case of JIA, as a result of immune suppressive/anti-inflammatory therapies.

### Transcription factor networks

**Fig. 4:**
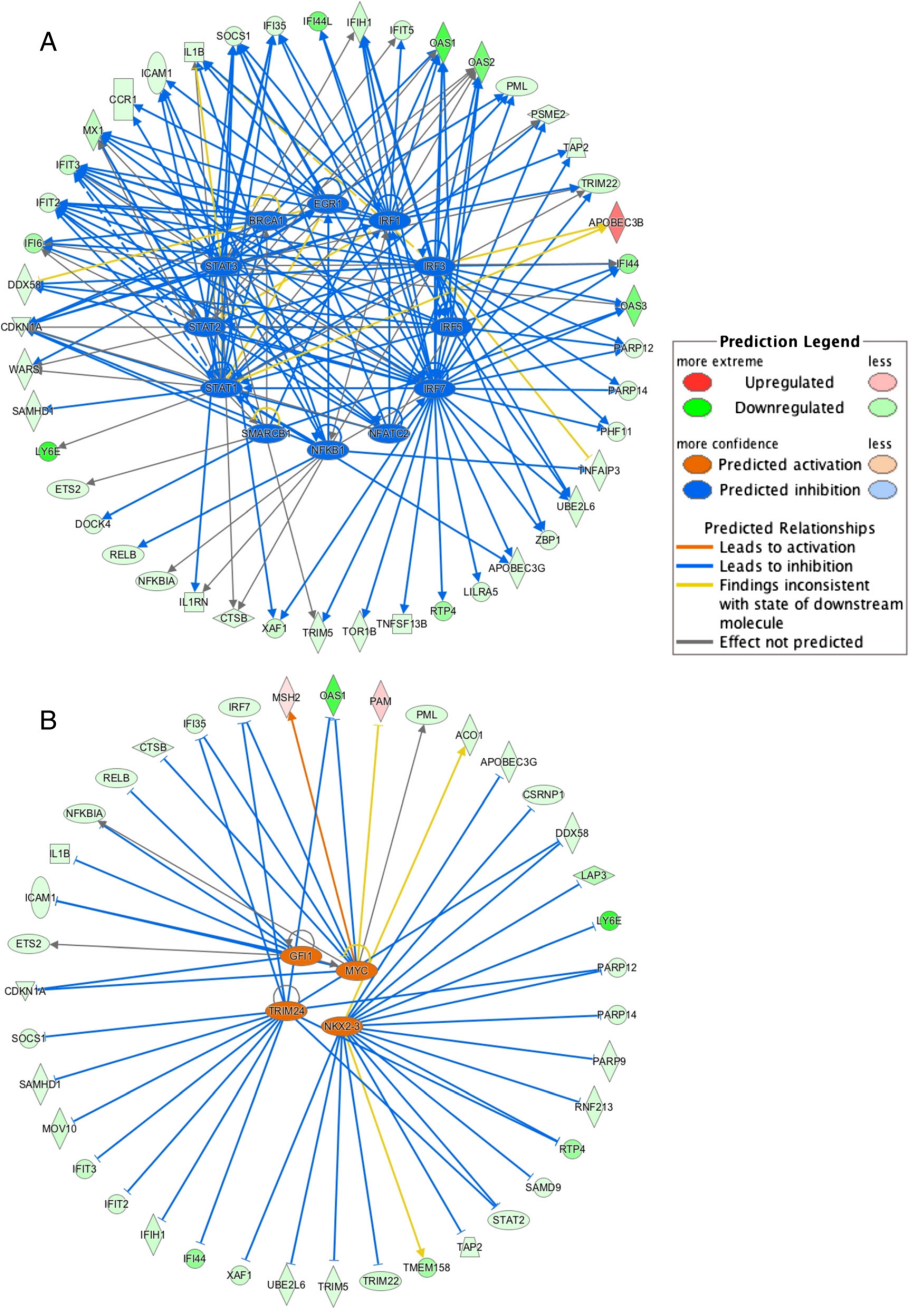
Transcription factors (inner circle) identified by the IPA software analysis for differentially expressed genes in the comparison of neutrophil RNAseq data between AD and CRM juvenile idiopathic arthritis patients. IPA analysis elucidated12 TFs that are predicted to be inhibited (**a**) and 4 TFs predicted to be activated (**b**). Each TF is connected with its target genes; dashed and solid connecting lines indicate an indirect or direct relationship with the master regulator respectively. The colors of the lines connecting TFs and their corresponding targets indicate the predicted activation (orange) or inhibition (blue) status

A long-term goal of our work is to understand how cellular networks are perturbed in chronic inflammatory diseases and the effects of therapy on restoring normal transcriptional wiring. We therefore undertook network analysis of RNA expression in the samples we had subjected to RNAseq. Upstream regulator analysis in IPA allowed the identification and determination of the state of activation of upstream regulators that might be responsible for the observed gene expression changes between different groups. We identified 2 transcription factors (TFs), GFI1 and NUPR1, from 131 differentially expressed genes between AD and CF, which were identified as being inhibited. GFI1 regulates genes *CDKN1A, EGR3, PCOLCE2, PLOD1* and *SPP1*; while NURP1 regulates genes FLVCR1, FUCA1, HBEGF, PLK3 and TMEM158. Analysis of the 159 genes differentially expressed between AD and CRM identified 121 TFs. Twelve of the 121 TFs (EGR1, BRCA1, IRF1, IRF7, IRF3, IRF5, NFATC2, NFKB1, SMARCB1, STAT1, STAT2 and STAT3) were identified as being inhibited (Fig. 4a), and 4 TFs (GFI1, MYC, NKX1-3 and TRIM24) were identified as activated (Fig. 4b). These TFs regulate more than 5 genes in a single overlapping regulatory network (Fig. 4). Signal Transducer and Activator of Transcription (STATs) have numerous functions in innate immunity [39, 40]. Specifically, STATs have key functions for neutrophils and regulate gene transcription by alternative proteolytic processing [41, 42]. NF-κB is also included is this network. The NF-κB pathway is induced by a wide variety of stimuli, including cytokines such as the tumor necrosis factor-alpha and interleukin-1β, both of which are the targets of biologic therapies used to treat rheumatoid arthritis and juvenile idiopathic arthritis [43, 44]. The involvement of the oncogenes MYC in gene regulation in JIA is consistent with what we have previously reported in gene expression analyses of neutrophils [26].

### Differential usage of exons

We applied DEXSeq [45] to test for differential exon usage in RNA-seq data. The DEXSeq analysis revealed that there were 204 significantly differential exon usages (*p* < 0.01) between AD and CRM (Additional file 6: Table S6). These differential exon usages were distributed in 179 genes. Of 179 genes demonstrating differential exon usage, 7 genes (*BTN3A2, IFI44L, IFIT3, LOXHD1, PAM, RNF213, SH3RF3*) also showed differential expression between AD and CRM at the gene level.

We also found 838 genes that demonstrated significant differential exon usage (*p* < 0.01) when we compared AD and CF (Additional file 7: Table S7). Differential exon usage was distributed in 678 genes. Of 678 genes demonstrating differential exon usage, 6 genes (*IFITM2, LOXHD1, MSH2,, RPH3A, SH3RF3, ZNF107*) also showed differential expression between AD and CF at the gene level.

The comparison of AD vs. CRM reveals a neutrophil response to therapeutic intervention and thus may be somewhat artificial. In contrast, the CF and AD situations are rather similar (chronic inflammation in soft tissues), and subtle re-organization of the transcriptome in these parallel but slight different scenarios is actually quite interesting.

### Differentially expressed lncRNA

As noted above, we identified 2981 lncRNAs expressed in neutrophils in at least one of nine subjects. We applied differential expression analysis of all the lncRNAs that were expressed in all the libraries using DESeq. The analysis revealed there are 38 lncRNAs that showed differential expression when the AD group was compared with CRM. There were 30 lncRNAs that showed detectable differences in expression between AD and CF. There were 63 DE lncRNAs in CRM vs CF comparison (Additional file 8: Table S8). These results show that lncRNA expression is disease and disease-state specific.

To validate the RNA-seq data, we chose randomly 7 of 38 DE lncRNAs between AD and CRM, and performed real-time PCR in an independent patient cohort. Table 1 shows that 7 lncRNAs differentially expressed in the RNA-seq analysis were also differentially expressed in real-time PCR. It is interesting to note that two of these lncRNA, lncIFITM-4 and lncPML-1, are adjacent to differentially-expressed genes (IFITM3 and PML, respectively), suggesting that these transcripts may act directly in to regulate gene expression in neutrophils and fine-tune transcriptional responses to specific inflammatory/disease states. As the functions of lncRNAs are largely unknown, an approach for inferring putative functions of long ncRNAs is to examine protein-coding genes located near ncRNAs of interest [46, 47]. We examined the expression pattern of paired neighbor protein-coding genes of differential expressed lncRNAs between AD and CRM. Interestingly, we found that six neighbor protein-coding genes (*CHSY1, IFITM3, LILRA5, PGM5, PML, ZCCHC2*) of these DE lncRNAs were also differentially expressed at same direction. Of these 5 genes, *IFITM3* mRNA and *PML* mRNA are known to be upregulated in labial minor salivary glands and associated with in primary Sjogren’s syndrome [48].

## Discussion

We have demonstrated the feasibility of preparing high-quality RNA from human neutrophils in sufficient quantity to perform RNA-Seq in the context of different human phenotypes. Furthermore, we have demonstrated that such studies can be undertaken even with the relatively small amounts of human blood available for translational studies in children. Finally, we demonstrate that neutrophil transcriptomes show subtle variations that correspond to specific human phenotypes and inflammatory conditions.

Neutrophils are a critical cell in human defense, and depletion of neutrophils from peripheral blood, whether as a consequence of therapeutic efforts or human disease, has almost immediate adverse consequences [49]. Furthermore, neutrophils play a critical role in their ability to direct and instruct the adaptive immune system [50]. Despite the importance of these cells, they are conspicuously absent from the both ENCODE and Roadmap Epigenomics data sets. Thus, we know little of the functional genomics of these cells and how neutrophil genomes adapt to regulate transcription in response to external signals and disease states.

Like most leukocyte genomes, neutrophils show substantial transcriptional complexity. Although most transcripts were expressed at low levels (compared with lymphocytes, for example) we found that more than 7700 genes, or about 30 % of all the known protein-coding genes, were detected in all nine samples. Furthermore, isoform usage was extensive, with more than 9500 detected in each of the three different phenotypes. While extensive RNA splicing is known to characterize adaptive immune responses [51], these findings suggest that even neutrophils carry the capacity for supple, threat-specific adaptations to host injury or infection. The latter point is corroborated by the subtle differences in the transcriptomes of the three different childhood phenotypes that we studied.

Because of the differences between the three different phenotypes, our studies suggest that RNAseq may be a substantial improvement over hybridization-based gene expression arrays for the development of informative biomarkers in human disease. As gene expression microarrays became widely available and affordable, there was considerable excitement about their use in developing predictive or diagnostic biomarkers [4]. It was disappointing, then, when biomarkers identified in one cohort (e.g., for prediction of response to therapy) showed little or overlap with biomarkers developed in independent cohorts [52, 53]. While there have been successful attempts to corroborate gene array data in independent patient cohorts [3], the limited dynamic range and considerable technical variation (large batch effects) of hybridization-based arrays will very likely continue to limit their utility for medical purposes. With next-gen sequencing costs continuing to fall, the possibility of developing “personalized transcriptomes” for diagnosis or prognosis (e.g., predicting therapeutic response) seems an achievable goal.

This sample size is too small to make broad inferences about the pathogenesis of JIA, although some interesting findings emerge from these data. For example, we have previously reported the involvement of interferon gamma in gene expression networks constructed from gene microarray data in JIA neutrophils [9]. The current data suggest an attenuation of type 1 interferon responses in JIA neutrophils, a new finding and not one that we have previously discerned in expression data in JIA neutrophils [1, 3, 26], although we see decreased expression of numerous pro-inflammatory genes in JIA neutrophils using this approach. These findings are probably medically relevant: a recent study shows that IFN-response gene expression levels in neutrophils in adult RA correlates with a good response to TNF inhibitor therapy [54]. While we believe that our findings support efforts to continue biomarker development from human neutrophils in chronic inflammatory diseases, it is unlikely that, by itself, neutrophil transcriptome profiling will not be sufficient to crisply elucidate the pathogenesis of JIA or other complex traits characterized by chronic, indolent inflammation.

RNAseq also allowed us to characterized the expression of lncRNAs in neutrophils in CF patients and JIA patients with different disease states. Our results demonstrate a large number of lncRNAs commonly expressed in neutrophils, and these data thus provide a useful resource for lncRNA expression in human neutrophils. We also analyzed differential expression analysis of the lncRNAs between diseases or disease states and identified 38 differentially expressed lncRNAs in AD vs CRM comparison, and 30 in AD vs CF. These results provide further evidence that neutrophils exhibit considerable adaptability in their transciptomes and that gene and transcript expression is disease or disease state specific.

## Conclusions

Human neutrophils exhibit surprising specificity in their transcriptional responses, which vary both between specific diseases and even with specific disease states. These findings were observed for genes, gene isoforms, and non-coding transcripts. Furthermore, our findings show that RNA sequencing may be a useful method for investigating the connections between gene/transcript expression and human phenotypes, including disease phenotypes.

## Additional files

Additional file 1: Table S1. Genes identified as expressed in neutrophils in all 9 subjects. (XLSX 893 kb) Additional file 2: Table S2. Gene ontology analysis of genes expressed in neutrophils. (XLSX 212 kb) Additional file 3: Table S3. Long non-coding RNAs identified as expressed in neutrophils. (XLSX 341 kb) Additional file 4: Table S4. Isoforms identified as expressed in neutrophils. (XLSX 4567 kb) Additional file 5: Table S5. Differentially expressed genes in different groups comparison. (XLSX 34 kb) Additional file 6: Table S6. Differential usage of exons in AD vs CRM comparison. (XLSX 25 kb) Additional file 7: Table S7. Differential usage of exons in AD vs CF comparison. (XLSX 103 kb) Additional file 8: Table S8. Differentially expressed long non-coding RNAs in different groups comparison. (XLSX 56 kb)
